# The Correlation between Waist Circumference and the Pro-Inflammatory Adipokines in Diabetic Retinopathy of Type 2 Diabetes Patients

**DOI:** 10.3390/ijms24032036

**Published:** 2023-01-20

**Authors:** Yeo Jin Lee, Joeng Ju Kim, Jongmin Kim, Dong-Woo Cho, Jae Yon Won

**Affiliations:** 1 Department of Ophthalmology and Visual Science, Eunpyeong St. Mary’s Hospital, The Catholic University of Korea, Seoul 03312, Republic of Korea; 2 Catholic Institute for Visual Science, College of Medicine, The Catholic University of Korea, Seoul 14662, Republic of Korea; 3 Department of Mechanical Engineering, Pohang University of Science and Technology (POSTECH), Pohang 37673, Republic of Korea

**Keywords:** diabetic retinopathy, central obesity, adipokines

## Abstract

Central obesity is one of the major risk factors for type 2 diabetes mellitus (DM), and the most common complication of DM is diabetic retinopathy. However, the exact relationship between obesity and DR remains unknown. In this study, we evaluate the effect of obesity on DR by comparing the aqueous humor-derived adipokines. For the analysis, 37 DR patients and 29 non-DR-patients participated. To evaluate the obesity of the patients, body mass index (BMI) and waist circumference (WC) were used. By comparing the concentrations of adipokines obtained from the aqueous humor of the two groups, the relationship between DR and adipokines was analyzed. In addition, by analyzing the correlation between obesity and adipokines in patients, the relationship between central obesity and DR was finally confirmed. The WC was significantly higher in patients than in the non-patient group. The concentrations of all adipokines compared in this study were significantly higher in the DR group than in the non-DM group (*p* < 0.05). Among them, adiponectin, leptin, TNF-α, Factor D (adipsin), lipocalin-2 (NGAL), Serpin E1 (PAI-1), and CXCL8 (IL-8) were confirmed to have a positive correlation with central obesity (defined as WC). These findings suggest that central obesity is strongly associated with the risk of DR.

## 1. Introduction

The number of diabetes patients has been increased in recent decades, and it is expected to rise to 592 million by 2035 [1]. The DM causes various kinds of complications in both the macrovascular, such as coronary artery disease, stroke and peripheral arterial disease, and microvascular, including retinopathy, nephropathy, and neuropathy.

Among them, diabetic retinopathy (DR) is one of the common complications of diabetes mellitus (DM) and is a frequent cause of blindness. The occurrence and progression of DR are closely linked to the duration of diabetes, chronic hyperglycemia, high blood pressure, pregnancy, puberty, kidney disease, and hyperlipidemia [2,3,4]. It is also well known that obesity is a major risk factor for type 2 diabetes [5], although it is unclear whether it is a risk factor for DR.

Several attempts have been made to explain the relationship between DR and obesity. There have been several studies analyzing the relationship between body mass index (BMI), waist-to-hip ratio (WHR), or waist circumference (WC) and severity of DR, and recently, there has been a meta-analysis [6,7,8,9,10,11,12,13,14].

Moreover, some studies can infer the relationship between them. Banks at el. [15] reported that the progression of DR decreased after Roux-en-Y gastric bypass surgery, and a meta-analysis of physical activity and DR risk confirmed that physical activity was associated with low DR risk [16]. For these reasons, we tried to clarify the ambiguous relationship between central obesity and DR through molecular-level analysis.

The term adipokines refers to peptides or proteins secreted from adipocytes in order to maintain metabolic homeostasis. Adipokines are categorized into ‘pro-inflammatory adipokines’ and ‘anti-inflammatory adipokines’. The former includes leptin, TNF, IL-6, resistin, retinol-binding protein 4 (RBP4), lipocalin 2, IL-8, adipsin, angiopoietin-like protein 2 (ANGPTL2), CC-chemokine ligand 2 (CCL2), MCP-1, PAI-1, and CXC-chemokine ligand 5 (CXCL5). The latter includes adiponectin and sFRP5 [17,18,19,20,21]. In obese patients, levels of pro-inflammatory adipokines rise, while anti-inflammatory adipokines decrease. This results in raised inflammatory response and insulin resistance, increasing the incidence of type 2 diabetes and/or cardiovascular disease [18,22,23].

Changes in adipokine levels in the plasma of obese patients and those with type 2 diabetes have been reported in numerous previous studies. There have also been studies on the concentrations of adiponectin and leptin in the aqueous humor of DR patients [24,25]. However, to the best of our knowledge, no studies have been conducted on the association of DR with central obesity by measuring the WC and analyzing the correlation with these adipokines; therefore, we tried to confirm them in the present work.

Additionally, inflammation and angiogenesis play an important role in the pathogenesis of DR, and several previous studies have demonstrated an increase in related biomarkers in aqueous humor [26,27]. Inflammatory cytokines are also increased in obese patients. In addition, some studies have shown that adipokines are associated with angiogenesis [28]. Therefore, we further analyzed the concentrations of inflammatory cytokines and several growth factors in the aqueous humor of DR.

## 2. Results

### 2.1. Participants’ Characteristics

**Figure 1:**
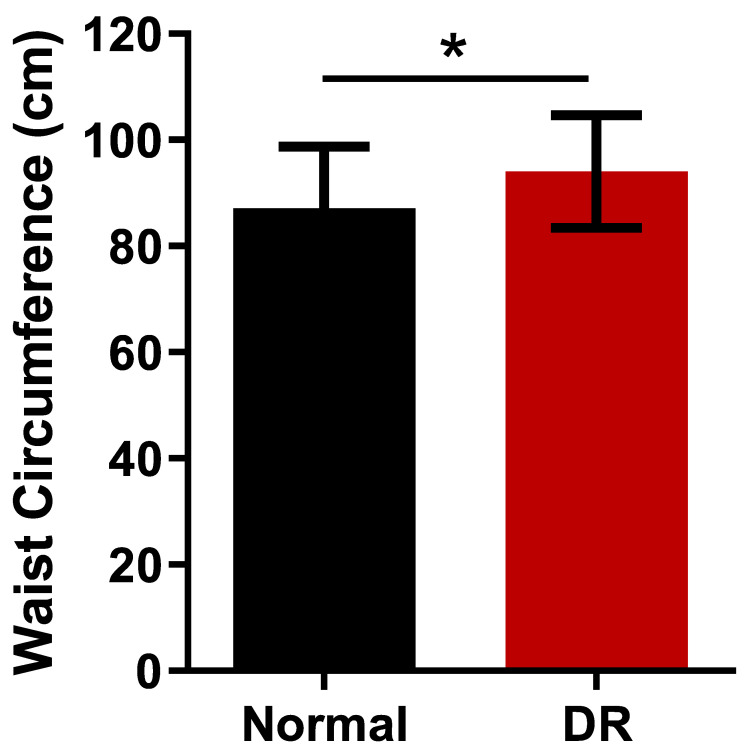
Waist circumferences between the normal and patients with DR. The data were compared using Student’s *t*-test and differences were considered significant when, * for *p* < 0.05.

Table 1 summarizes the baseline characteristics of all participants. Overall, 37 patients with DR (22 severe non-proliferative DR, and 15 proliferative DR) and 29 patients without DM (normal) were included in the study. HbA1C (5.60 ± 0.40% vs. 8.23 ± 1.59%, *p* < 0.001), weight (61.68 ± 14.53 kg vs. 68.16 ± 10.93 kg, *p* = 0.043), WC (87.57 ± 11.55 cm vs. 94.30 ± 10.53 cm, *p* = 0.016, and Figure 1), and BMI (25.00 ± 3.63 kg/m^2^ vs. 27.04 ± 3.46 kg/m^2^, *p* = 0.023) were significantly higher in the DR group than in the normal group. In sub-group analysis of DR by sex, WC is 103 ± 11.42 cm (male) and 89.23 ± 4.15 cm (female). In sub-group analysis of DR by severity, there is no statistically significant difference in WC and DM duration between severe NPDR and PDR (*p* = 0.125, *p* = 0.089). The DR group was significantly younger than in the normal group (60.30 ± 13.35 years vs. 68.52 ± 9.94 years, *p* = 0.020). The BCVA were 0.19 ± 0.32 logMAR and 0.59 ± 0.54 logMAR in the DR group and normal group, respectively and they were significantly lower in the DR group. There was no significant difference in the height and sex between the DR and normal group (*p* = 0.308, *p* = 0.310, respectively).

### 2.2. Biomarker Analysis in Aqueous Humors

**Figure 2:**
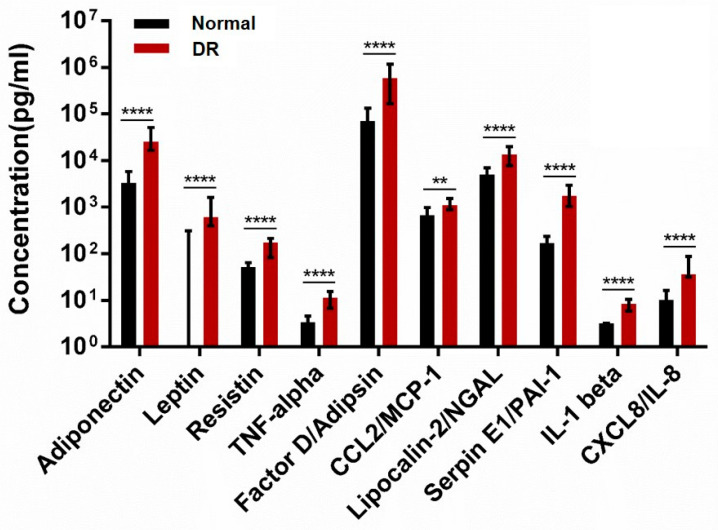
Adipokines levels between the normal and DR groups. The data were compared using Student’s *t*-test and differences were considered significant when, ** for *p* < 0.01., **** for *p* < 0.0001.

**Figure 3:**
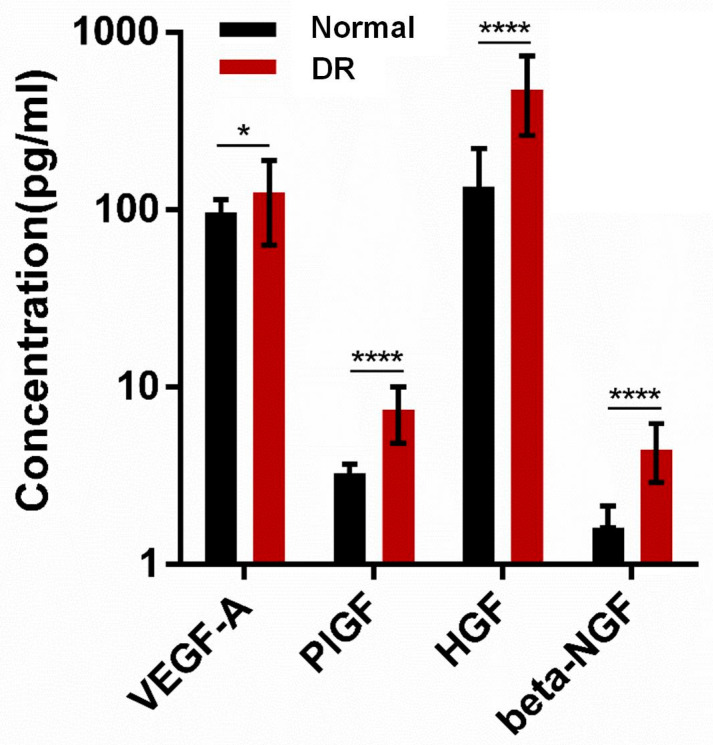
Growth factors levels between the normal and DR groups. The data were compared using Student’s *t*-test and differences were considered significant when, * for *p* < 0.05, **** for *p* < 0.0001.

Adiponectin, leptin, resistin, TNF-α, Factor D (adipsin), CCL2 (MCP-1), lipocalin-2 (NGAL), Serpin E1 (PAI-1), IL-1β, and CXCL8 (IL-8) were significantly higher in the DR group than in the normal group (*p* < 0.0001, *p* < 0.0001, *p* < 0.0001, *p* < 0.0001, *p* < 0.0001, *p* < 0.01, *p* < 0.0001, *p* < 0.0001, *p* < 0.0001 and *p* < 0.0001 respectively, Figure 2). In sub- group analysis of DR, there are no statistically significant differences in pro- inflammatory adipokines between severe NPDR and PDR. Adiponectin, leptin, resistin, TNF-α, Factor D (adipsin), CCL2 (MCP-1), Lipocalin-2 (NGAL), Serpin E1 (PAI-1), IL-1β, and CXCL8 (IL-8) were significantly different in the DR group than in the normal group (*p* = 0.067, *p* = 0.143, *p* = 0.052, *p* = 0.076, *p* = 0.153, *p* = 0.114, *p* = 0.051, *p* = 0.147, *p* = 0.079 and *p* = 0.117 respectively, Figure S1). Figure 3 shows that growth factors (VEGF-A, PIGF, HGF, and Beta-NGF) were significantly different between the two groups (*p* = 0.0436, *p* < 0.001, *p* < 0.001, and *p* < 0.001, respectively).

**Figure 4:**
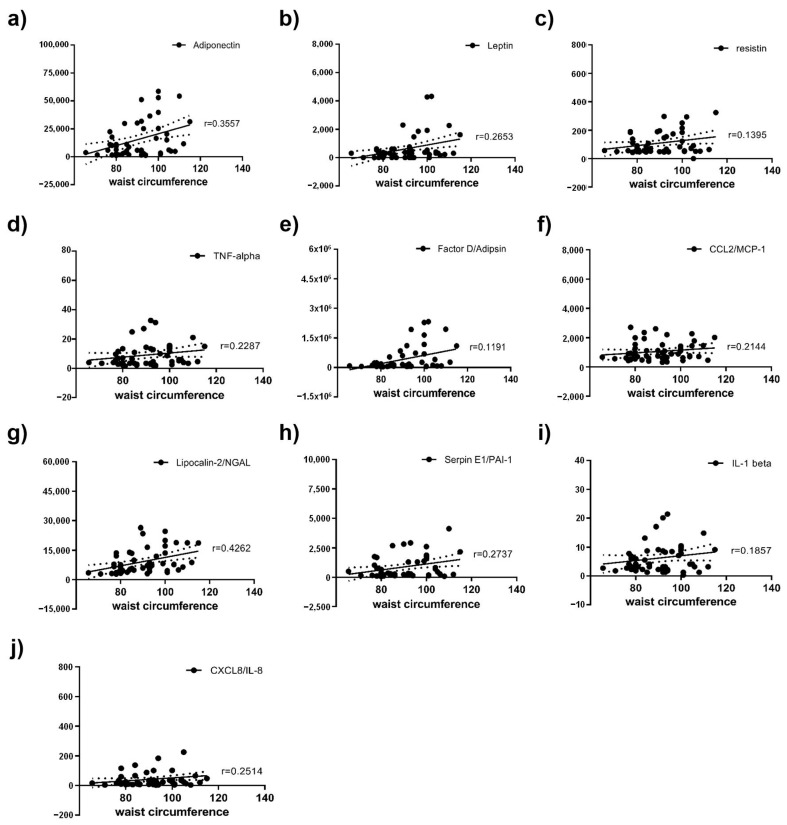
Scatterplots of correlations of WC and adipokines, including (**a**) adiponectin, (**b**) leptin, (**c**) resistin, (**d**) TNF-α, (**e**) Factor D/adipsin, (**f**) CCL2/MCP-1, (**g**) lipocalin-2/NGAL, (**h**) Serpin E1/PAI-1, (**i**) IL-1β, and (**j**) CXCL8 (IL-8) in the overall groups.

Spearman correlation between height, weight, WC, BMI, and adipokines was performed in all patients. Furthermore, Spearman correlation between adipokines and DM duration was performed. Adiponectin, leptin, resistin, TNF-α, Factor D (adipsin), CCL2 (MCP-1), lipocalin-2 (NGAL), Serpin E1 (PAI-1), IL-1β, and CXCL8 (IL-8) were significantly positive correlated with WC (Figure 4). Each of them had a positive correlation with WC (Spearman r = 0.3557, 0.2653, 0.1719, 0.2287, 0.3632, 0.2144, 0.4262, 0.2698, 0.2145, and 0.2514, respectively). In sub-group analysis of DR, adiponectin, leptin, resistin, TNF-α, Factor D (adipsin), CCL2 (MCP-1), lipocalin-2(NGAL), Serpin E1 (PAI-1), IL-1β, and CXCL8 (IL-8) in severe NPDR were significantly positively correlated with WC. Each of them had a positive correlation with WC (Spearman r = 0.3022, 0.2563, 0.143, 0.1654, 0.3317, 0.2042, 0.3727, 0.2078, 0.1826 and 0.2292, respectively). Adiponectin, leptin, resistin, TNF-α, Factor D (adipsin), CCL2 (MCP-1), lipocalin-2(NGAL), Serpin E1 (PAI-1), IL-1β, and CXCL8 (IL-8) in PDR were significantly positively correlated with WC. Each of them had a positive correlation with WC (Spearman r = 0.412, 0.274, 0.202, 0.288, 0.3934, 0.2236, 0.458, 0.339, 0.2482 and 0.274, respectively). On the other hand, BMI was only correlated with leptin (Spearman r = 0.244), and height, DM duration and weight had no correlation with any adipokines.

## 3. Discussion

Several studies have reported that central obesity is associated with DM, and some recent studies have concluded that it is also associated with DR. However, the exact cause of the relationship remains unknown.

DR has complex pathogenesis, including several mechanisms such as vascular, inflammatory, and neuronal signal. Among them, the inflammatory mechanism has been widely studied as molecular and structural alterations were observed [29]. Actually, some studies found that some pro-inflammatory cytokines were elevated in the serum of patients with DR [30,31]. The other previous studies have demonstrated changes in the concentration of inflammatory cytokines (CRP, TNF-α, IL-6, IL-1β, IL-8, cell adhesion molecules (CAMs), Serpin E1 (PAI-1), and RBP4) and angiogenic mediators (VEGF, PGF, PEGF, IGF-1, transforming growth factor beta, basic fibroblast growth factor, and HGF) in aqueous humor of DR [32]. Like previous studies, pro-inflammatory adipokines (resistin, TNF-α, Factor D (adipsin), CCL2 (MCP-1), lipocalin-2 (NGAL), Serpin E1 (PAI-1), IL-1β, and CXCL8 (IL-8)) concentrations in the aqueous humor of DR patients were found to be significantly higher than those of non-DM patients in our study. Growth factors (Beta-NGF, HGF, PIGF, and VEGF-A) also were found to be significantly higher than those of non-DM patients in our study. Although DR itself is related to angiogenesis, some studies have shown that inflammation-related adipokines also induce angiogenesis by deteriorating vascular endothelial cell function and consequently altering the production of endothelial mediators [28]. It is presumed that this also affected the increase in the growth factors’ concentrations.

Unlike other pro-inflammatory mechanisms, adipokines have a positive role in preventing diabetic complications by suppressing insulin resistance and controlling inflammation. Actually, adiponectin is maintained at high levels in the serum of healthy individuals. However, in our study, adiponectin concentration was increased in the aqueous humor of DR despite being an anti-inflammatory adipokine. Although the adiponectin concentration in the serum of obese patients is low, its concentration in the serum of DR patients is controversial.

Kuo et al. [33] showed the elevation of adiponectin level in DR patients’ serum and positive correlation with severity of the DR, and Yilmaz et al. [34] reported that serum adiponectin concentrations are lower in patients with type 2 diabetes and that these concentrations are associated with the severity of DR. However, adiponectin concentrations in the aqueous humor of DR are consistently high in most studies. Yang et al. [25] and Mao et al. [24] reported that the adiponectin levels in the aqueous humor of DR patients were higher than in controls, whereas the levels in their serum were not consistent between the two studies. They both proposed that the blood–retinal barrier (BRB) may play a key role in maintaining intra-ocular adiponectin homeostasis. In healthy individuals, aqueous humor adiponectin concentrations are much lower than serum adiponectin concentrations because of the BRB. They explained the aqueous humor adiponectin concentration increased due to increased BRB permeability as DR progressed. In addition, there was a study that aqueous humor leptin concentration was high in DR patients, and the present study showed the same results [25].

As a next step, we investigated whether the levels of adipokines and growth factors in the aqueous humor of DR patients is correlated with the degree of obesity. Parameters for measuring obesity include BMI, WHR, and WC. BMI is a method of measuring general obesity, and WC and WHR are methods of measuring central (abdominal) obesity. General obesity does not distinguish between fat and muscle, thus failing to reflect the higher body fat percentage of women compared with men and of older people compared with younger people. Therefore, it is accepted that measuring central obesity (abdominal obesity) using WHR or WC better reflects metabolic status, with high levels indicating a strong correlation with cardiovascular disorders [32]. There are other methods to measure visceral fat using bioelectrical impedance analysis (BIA) and computed tomography (CT) for more accurate fat measurement, but WHR and WC are more often used in order to reduce time and cost.

Recently, several studies have evaluated whether BMI, WC, and WHR as measurement methods of obesity were related to DR. The relationship between BMI and DR is controversial. Whereas some previous studies have reported a positive correlation [6,7,8], others found no relationship or a negative correlation [9,10,11]. In addition, a recent meta-analysis study has concluded that no relationship exists between BMI and DR [12]. They explained that general obesity had protective and adverse effects on the risk of DR.

On the other hand, some studies have found that WC and WHR are significantly related to DR. Central obesity is known to contribute to insulin resistance and inflammation, which is an important pathophysiology for both DM and DR [13]. Therefore, WC and WHR are thought to be more sensitive markers than BMI with regard to indicating changes in metabolic state, and they are also thought to be more relevant to DR. Man et al. [11] reported that a greater WHR is associated with the increased likelihood and severity of DR in type 2 diabetes, whereas a higher BMI is inversely associated with the presence and severity of the disease. However, WC is the simplest method to measure the central obesity. WC is a more suitable method to measure the abdominal obesity compared to WHR, which could be underestimated in obese patients because of the high hip circumference of the denominator. Sometimes, it is difficult in clinical settings to obtain an accurate measurement of hip circumference for WHR as compared to WC. There is no evidence that WHR is a more appropriate method than BMI or WC to measure the central obesity in systemic review study. BMI and WC might still be suitable tools for measuring for central obesity [35,36]. Actually, a study conducted on the Chinese population indicated that central obesity, particularly as defined by WC, was associated with the risk of DR [14]. Thus, we use the WC and BMI for measuring central obesity. In our study, BMI was correlated with leptin only, and WC was found to have a significant correlation with pro-inflammatory adipokines (leptin, resistin, TNF-α, Factor D (adipsin), CCL2 (MCP-1), lipocalin-2 (NGAL), Serpin E1 (PAI-1), IL-1β, and CXCL8 (IL-8)). These results could clarify the ambiguous relationship between central obesity and DR, as shown in previous studies, and suggest a strong relationship between central obesity and DR.

The present study had several limitations. First, we could not identify patient’s underlying diseases such as high blood pressure, hyperlipidemia, and cardiovascular disease that could affect cytokines. Among these underlying diseases, dyslipidemia, which could affect adipokines especially, should be further evaluated. Actually, 19 patients of the normal group and 31 patients of the DM group are being treated for dyslipidemia in our study. So, we could not analyze the relationship between dyslipidemia and this study. Second, we also could not collect information on DR severity; hence, we were unable to measure the change in the concentration of cytokines based on DR severity. The correlation between obesity and DR progression was also unknown. There is no comparison between obesity and not obese in DM patients. Thus, in order to elucidate the diverse and accurate relationship between central obesity and DR, further research on this is needed, and a study providing a comparison with non-DR patients (with DM) or serum adipokine concentration is required.

Nevertheless, the strength of this study lies in the fact that it is the first to confirm the possibility of the relationship between DR and central obesity by directly measuring adipokines in the aqueous humor and analyzing the correlation. We hope that these findings could help with DR treatment.

## 4. Materials and Methods

### 4.1. Study Population

This study was approved by the Institutional Review Board/Ethics Committee of the Catholic University of Korea and performed in accordance with the tenets of the Helsinki Declaration. Informed consent was obtained from all patients before they enrolled in the study (2021-3384-0002).

We recruited 37 patients with DR (DR group) and 29 non-DM patients (normal group) who were undergoing cataract surgery or intraocular injection at Eunpyeong St. Mary’s Hospital in Korea with type 2 DM from February 2021 to April 2022.

Patients with the following conditions were excluded from this study: any ocular disease except cataract or DR, previous intraocular surgery or intraocular injection or laser therapy including panretinal photocoagulation. The patients were also excluded if there was any complication during cataract surgery or intraocular injection.

Comprehensive medical and ophthalmic histories were collected at the initial visit. Before cataract surgery or intraocular injection, height, weight, WC, and HbA1C were measured, and a comprehensive ophthalmic evaluation including best-corrected visual acuity (BCVA), fundoscopy, and optical coherence tomography (DRI OCT triton, Topcon Corporation, Tokyo, Japan) were also performed. DR was diagnosed by one ophthalmologist specialized in retina through a fundus photograph reading.

### 4.2. Sample Collection

Overall, 34 samples were obtained aqueous humor during cataract surgery and 32 were obtained during intraocular injection. Before the cataract surgery or intraocular injection, an anterior chamber paracentesis was performed, and 0.05–0.1mL of aqueous humor was collected using a 1 mL syringe to control intraocular pressure. The samples were immediately deposited into a sterile plastic tube and stored at −80 °C until sample analyses.

### 4.3. Analysis of Cytokines in Aqueous Humor and Serum Samples

Concentrations of adiponectin, leptin, resistin, TNF-α, Factor D (adipsin), CCL2 (MCP-1), lipocalin-2 (NGAL), Serpin E1 (PAI-1), IL-1β, CXCL8 (IL-8), VEGF-A, PIGF, HGF, and Beta-NGF in aqueous humor from the anterior chamber of patients in Table 1 were measured. The sample was melted immediately before the experiment, vortexed, and centrifuged, and the supernatant was diluted 1/2 before use. For minimizing the error, all measurements were conducted in triplicate. The reaction between the sample and the bead–Ab mixture was performed at room temperature for 2 h. Analyte-specific antibodies were pre-coated onto magnetic microparticles embedded with fluorophores at set ratios for each unique microparticle region. The immobilized antibodies bound to the analytes of interest. A biotinylated antibody cocktail specific to the analytes of interest is added to each well. In addition, streptavidin–phycoerythrin conjugate (Streptavidin-PE), which binds to the biotinylated antibody, was added to each well. A Luminex^®^ analyzer (Luminex, Austin, TX, USA) was used for detection. The Luminex analyzer flows through the beads one-by-one, reads the antibody type, and measures the median fluorescence intensity (MFI). It is a technique that allows the analysis of many samples in a single reaction and a multiplexed microsphere suspension immunoassay that detects and quantitates unique microspheres attached to specific antibodies.

### 4.4. Measurements of Obesity

BMI was calculated as weight divided by the square of height (kg/m^2^). WC was measured at the narrowest point between the last floating rib and the iliac crest (cm) [37]. We used WC as a measure of central obesity.

### 4.5. Statistical Analysis

Statistical analysis was performed using SPSS Statistics version 26.0 (SPSS Inc., Chicago, IL, USA). For comparison of two groups, continuous variables were compared using unpaired *t*-test or Mann–Whitney U test and categorical variables using chi-squared test. Spearman’s rank-order correlation coefficients were used to determine the relationships between the height, weight, WC, BMI, and adipokine levels. A *p*-value <0.05 was considered statistically significant.

## 5. Conclusions

The present study directly measured aqueous humor adipokines, which were significantly higher in the DR group. Furthermore, our study showed the positive correlation between concentrations of adipokines and WC. These results suggest that central obesity is an important factor in the development of DR.

## Figures and Tables

**Table 1:** Baseline demographic and clinical characteristics of the study participants.

	Normal	DR	*p*-Value
N, eyes	29	37	-
Age (years)	68.52 ± 9.94	60.30 ± 13.35	0.020 *
Sex, male (%)	9 (31.0%)	16 (43.2%)	0.310 ^†^
DM duration (mon)	0	219.57 ± 130.58	-
HbA1c (%)	5.60 ± 0.40	8.23 ± 1.59	<0.001 *
Total Cholesterol (mg/dL)	178.0 ± 33.41	191.5 ± 47.56	0.2320 ^‡^
Triglycerides (mg/dL)	100.1 ± 58.97	126.2 ± 55.23	0.5336 ^‡^
HDL-C (mg/dL)	50.2 ± 10.78	46.4 ± 12.57	0.2366 ^‡^
LDL-C (mg/dL)	107.9 ± 29.64	117.5 ± 31.54	0.1243 ^‡^
BCVA (logMAR)	0.19 ± 0.32	0.59 ± 0.54	<0.001 *
Height (cm)	156.17 ± 10.45	158.67 ± 9.25	0.308 ^‡^
Weight (kg)	61.68 ± 14.53	68.16 ± 10.93	0.043 ^‡^
WC (cm)	87.57 ± 11.55	94.30 ± 10.53	0.016 ^‡^
BMI (kg/m^2^)	25.00 ±3.63	27.04 ± 3.46	0.023 ^‡^

Data are expressed as mean ± standard deviation (95% confidence interval). Abbreviations: DR, diabetic retinopathy; BCVA, best corrected visual acuity; logMAR, logarithm of the minimum angle of resolution; WC, waist circumference; BMI, body mass index * Mann–Whitney test; ^†^ Chi-square test; ^‡^ Student’s *t*-test.

## Data Availability

Not applicable.

## Supplementary Materials

The following supporting information can be downloaded at: https://www.mdpi.com/article/10.3390/ijms24032036/s1.
