# Supplementary figures and images for: The Correlation between Waist Circumference and the Pro-Inflammatory Adipokines in Diabetic Retinopathy of Type 2 Diabetes Patients
Source: Int J Mol Sci. 2023 Jan 20;24(3):2036. doi: 10.3390/ijms24032036 (PMC9917192; doi:10.3390/ijms24032036)

## Supporting Information

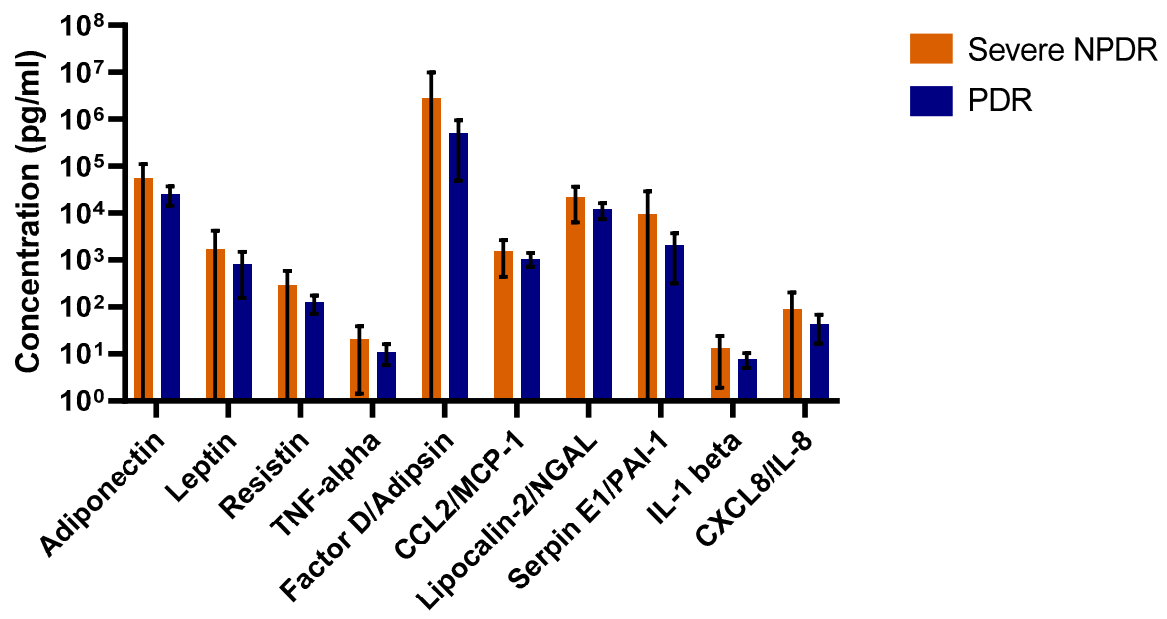

Figure S1. Adipokines levels between the Severe NPDR and PDR groups.

Supplement: Supplementary file 1 [file ijms-24-02036-s001.zip › ijms-2017410-supplementary.pdf]
